# Postpartum Pulmonary Hypertension Masquerading as Submassive Pulmonary Embolism: A Case Report and a Literature Review

**DOI:** 10.1155/2020/8899562

**Published:** 2020-06-22

**Authors:** Mahmood Mubasher, Amir Hanafi, Tausif Syed, Abir Zinal, Ibrahim Y. Abubeker, Mouhand F. H. Mohamed, Mohan Rao, Ryan Hoefen, Mohammed Danjuma

**Affiliations:** ^1^Rochester Regional Health-Unity Hospital, Rochester, NY, USA; ^2^Internal Medicine Department, Hamad General Hospital, Hamad Medical, Corporation, Doha, Qatar

## Abstract

Postpartum pulmonary hypertension (PPPHT) is an extremely rare disorder, with few reported cases. Late diagnosis and treatment are associated with significant morbidity and mortality. We present an 18-year-old female patient who presented four-week postpartum with a typical submissive pulmonary embolism picture subsequently diagnosed as postpartum pulmonary hypertension. The patient had an excellent response to treatment, with a dramatic improvement in her functional status. The authors aim to urge physicians to keep this rare disorder in mind as timely and accurate diagnosis is crucial for management—additionally, the importance of counseling patients about the imminent risks associated with planned future pregnancies.

## 1. Background

The sixth world symposium on pulmonary hypertension defines pulmonary hypertension (PHT) as the mean pulmonary artery pressure (mPAP) > 20 mmHg or pulmonary vascular resistance (PVR) > 3 Wood units [1]. Patients with PHT typically present with progressive fatigue and shortness of breath; this eventually leads to the development of right heart failure. PHT secondary to left heart disease (Type 2 PHT) continues to be the most common type of pulmonary hypertension [2, 3]. PPPHT is a rare variant of PHT. The period of the most considerable risk is within 30 days after delivery [4]. Clinical manifestations vary; presentation as acute massive pulmonary embolism was reported [4, 5]. Whilst its exact pathogenesis remains uncertain, the interaction of a host of factors may play a role in the evolution and progression of PPPHT. The most notable amongst these factors includes the role of cardiac output during pregnancy and delivery and autotransfusion from the uteroplacental circulation to the systemic circulation, along with the release of uterine pressure on the inferior vena cava after delivery suddenly increasing the preload [6, 7]. The second factor is a possible underlying placental hypoxia that induces the release of biological factors that impact on vascular remodeling and vasoconstriction through their effect on the endothelium and smooth muscles [8]. Thirdly (although not supported by robust evidence), amniotic fluid embolism during labor that inadvertently reached the pulmonary circulation could potentially induce an inflammatory reaction that leads to an increase in pulmonary vascular resistance [9]. Recent advances in clinical therapeutics of PTH have seen a significant reduction in maternal mortality attributable to it from 30-50% to 17-30% [10].

## 2. Case Presentation

An 18-year-old female patient with a past medical history significant for iron deficiency anemia presented four-week postpartum (first pregnancy) with a one-week history of chest pain and shortness of breath. The patient described a left-sided chest pain as not radiating, constant and stabbing in nature, moderate to severe, pleuritic, and not related to physical activity. The pain was associated with worsening shortness of breath and palpitations. The patient reported associated exertional dyspnea, dizziness, and fatigue that restricted her ability to take care of her baby. She denied fever, orthopnoea, paroxysmal nocturnal dyspnea, or lower limb swelling. The patient had recently returned from a twenty-eight-hour car journey. Apart from anemia, her pregnancy and delivery were both uneventful. The patient has no allergies, no family history of cardiovascular, or clotting disorders.

On examination, the patient was distressed, tachycardic, and tachypneic and had normal blood pressure. She required FiO_2_ of 44% to maintain normal saturation. Heartbeat was regular: soft heart sounds without murmurs, gallops, or rubs. There was no parasternal heave and no clinical signs of fluid overload. Moreover, there were no clinical features of endocarditis. She had normal vesicular breath sounds bilateral, with no crackles or wheezes. Lower limb examination was normal without signs of deep vein thrombosis.

**Figure 1:**
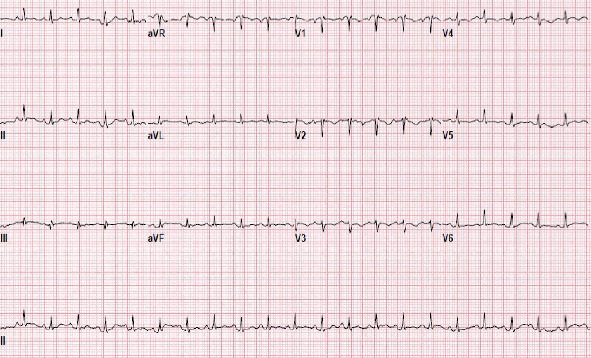
Electrocardiogram of the patient showing sinus tachycardia with T wave inversion in leads V1-3.

Her EKG showed sinus tachycardia with T wave inversion in leads V1-3 (Figure 1).

**Figure 2:**
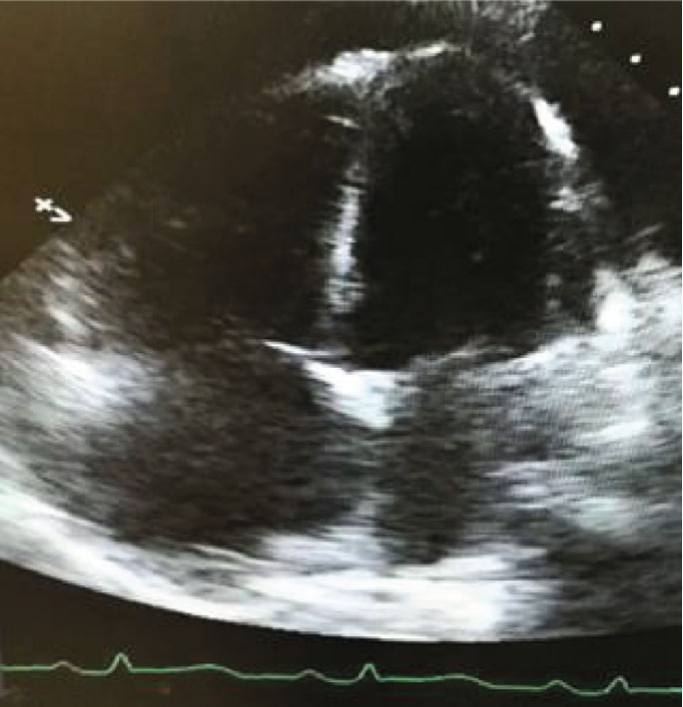
Transthoracic echocardiogram showing marked dilatation of right ventricle with globally reduced right ventricular systolic function.

**Figure 3:**
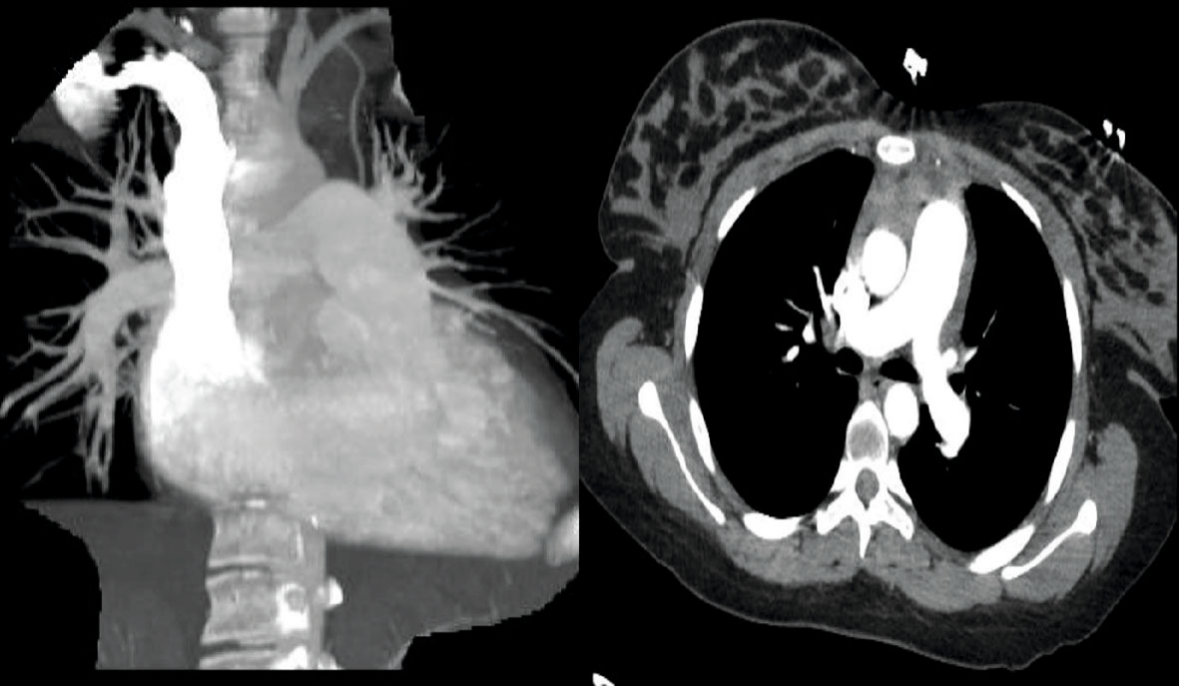
CT pulmonary angiogram showing dilated pulmonary trunk, with no evidence of pulmonary embolism.

Biochemical investigations showed a D-dimer of 3331 ng/ml (<500 ng/ml), BNP of 1034 pg/ml (<125 pg/ml), mildly elevated troponin I, and microcytic anemia. Otherwise, renal function, electrolytes, liver function tests, and thyroid hormones were within normal limits. Transthoracic echocardiogram showed a severely dilated right ventricle with globally reduced right ventricular systolic function and a left ventricular ejection fraction of 45-50% (Figure 2). CT pulmonary angiogram showed a dilated pulmonary trunk with no evidence of pulmonary embolism (Figure 3). An extensive venous thromboembolic workup including ultrasound Doppler of both lower limbs, CT venogram of the pelvis and lower limbs, and V/Q scan was negative.

Cardiac MRI showed a severely dilated right ventricle with a strain pattern with significant global reduced right ventricular systolic function, no signs of dysplasia or myocarditis, normal coronary anatomy, and no septal defects. Coronary angiography showed no coronary artery disease or dissection. Right heart catheterization (RHC) revealed the following: right atrial pressure = 12 mmHg (normal range 1-8 mmHg), right systolic ventricular pressure = 45 mmHg (normal range 15-30 mmHg), mean pulmonary artery pressure = 31 mmHg (normal range 10-22), pulmonary capillary wedge pressure = 7 mmHg (normal range 6-15), cardiac output = 2.97 L/min (normal range00204-8 mmHg), and cardiac index of 1.74/min/m^2^ (normal range 2.8-4.2), respectively.

Eventually, the patient was diagnosed with postpartum pulmonary hypertension. She was admitted and treated in the intensive care unit under the direct care of a pulmonary hypertension team. Her management was guided by a close follow-up of her hemodynamics. Given the high risk of sudden deterioration at any given point, inotropic support was considered and planned. Luckily, the patient's blood pressure remained normal. Good perfusion pressure was supported by normal lactate measures and absence of end-organ damage. Intravenous furosemide infusion was initiated with close monitoring of volume status, which was well tolerated without the need for inotropic support along with parenteral iron. The patient was discharged on torsemide and spironolactone.

## 3. Outcome and Follow-Up

On review three weeks postdischarge, her functional status was back to premorbid levels with improved exercise tolerance. Repeated transthoracic echocardiogram revealed no significant interval changes. Repeat right heart catheterization showed mild pulmonary hypertension, normal right and left ventricular filling pressures, and normalization of the cardiac output and index. The patient was counseled about the importance of contraception and the risks of pregnancy, given the associated mortality risk. However, a few months later, the patient became pregnant, and after a multidisciplinary meeting and recounseling, she underwent a therapeutic abortion. She remains to follow in the pulmonology clinic and is doing well.

## 4. Discussion

Postpartum pulmonary hypertension is an infrequent yet serious entity that can easily be overlooked. Keeping it in mind is very important as early diagnosis and appropriate treatment can have a significant impact on the patient's morbidity and mortality.

The presentation of our patient's submassive pulmonary embolism picture bore a resemblance to other published reports [4, 5]. As was the case with our patient, an extensive workup is usually advised to rule out venous thromboembolism. According to the PIOPED II trial [11], the sensitivity of CT pulmonary angiogram (CTPA) in this regard is about 83%. With high pulmonary embolism probability, the negative predictive value is 95% and increases to 97% if combined by negative CT venogram [11]. Even though amniotic fluid embolism is commonly seen during or immediately after labor, V/Q scan was done to rule it out as the diagnostic yield of CTPA with regard to amniotic fluid embolism is very low. Our patient had sex during the puerperium, which may have made her at risk of having air embolism, in which the penis is thought to act as a piston forcing air into the vagina [12–14]. However, the absence of air in echocardiogram made this unlikely. Coronary artery disease and dissection were ruled out by coronary angiography. Cardiac MRI ruled out myocarditis and arrhythmogenic right ventricular dysplasia as well as the unroofed coronary sinus. Right heart catheterization (RHC) confirmed the PHT as the primary cause of right heart strain, and the normal pulmonary capillary wedge pressure excluded a left ventricular cause of the PHT. The low cardiac output and low cardiac index are likely secondary to the decreased preload secondary to PHT.

It is difficult to know whether the pregnancy and labor-induced stress unmasked a preexisting PHT or if this is a new onset PHT. Owing to the absence of preexisting PHT in our patient, the lack of prior symptoms, the event-free pregnancy, and the start of symptoms three-week postpartum, we strongly believe that PPPHT is the correct diagnosis. Patients with PPPHT should be treated in intensive care units under the care of a pulmonary hypertension specialist team. Patients may require supplementary oxygen to maintain adequate oxygenation. Fluid overload should be treated with diuretics; however, the presence of right heart failure (RHF) makes treatment challenging. Patients often require an invasive assessment of hemodynamics to prevent loss of the preload the patient depends on to maintain cardiac output. Our patient had a dramatic improvement of symptoms and normalization of functional status with diuretics alone without the need for pulmonary hypertension-targeted treatments. Treatment of iron deficiency may help in controlling PHT, as previously reported in several studies, which showed that iron deficiency in patients with PHT was considered an important contributing factor [15].

In refractory cases, balloon atrial septostomy is considered a bridge for lung transplantation as it increases preload, cardiac output, and systemic oxygen transport [16]. However, this should be done in a stepwise manner to avoid refractory hypoxemia that may increase mortality [17, 18]. Lung transplant is indicated in the World Health Organization (WHO) functional class III or IV during escalating therapy, rapidly progressive disease, and the continuous need for parenteral targeted pulmonary hypertension therapy. The average five-year survival posttransplantation is around 45-50% [19]. Although the overall progression and prognosis of PPPHT are not well understood, dramatic response to diuretics, lack of need of parenteral targeted pulmonary hypertension therapy, and the normalization of functional status are all favorable prognostic factors.

Even in patients with a good prognosis, future pregnancies are contraindicated. Advice on effective contraceptive methods must be given. Progestogen-only methods and intrauterine devices can be used in women with PHT. Injectable progesterone-only methods and estrogen-containing contraceptives may increase the risk of venous thromboembolism [6, 20, 21]. Dual contraception is indicated in women taking Bosentan due to the interaction between the drug and progesterone-based methods of contraception [6, 16]. Although our understanding of the progression and prognosis of PPPHT is limited, the tough decision of therapeutic abortion was taken after a multidisciplinary meeting and a detailed discussion with the patient and was supported by the current pulmonary artery hypertension guidelines that recommend avoidance of pregnancy and termination of it if it happened [16].

## 5. Limitation

It is difficult to ascertain or conclude whether the pregnancy and labor-induced stress unmasked a preexisting PHT or if this is a new onset PHT. However, the favorable response to treatment is in keeping with new-onset PPPHT.

## 6. Conclusions

Postpartum pulmonary hypertension is an extremely rare disorder that has a significant impact on patients' morbidity and mortality. Early diagnosis and appropriate management under the direct supervision of pulmonary hypertension specialists are essential. Postpartum pulmonary hypertension is considered a relative contraindication for further pregnancies. Clinicians should counsel patients on adopting proper contraceptive plans.

## Data Availability

Data are available upon reasonable request.

## References

[1] Simonneau G., Montani D., Celermajer D. S. (2019). Haemodynamic definitions and updated clinical classification of pulmonary hypertension. *European Respiratory Journal*.

[2] Simonneau G., Robbins I. M., Beghetti M. (2009). Updated clinical classification of pulmonary hypertension. *Journal of the American College of Cardiology*.

[3] Oudiz R. J. (2007). Pulmonary hypertension associated with left-sided heart disease. *Clinics in Chest Medicine*.

[4] Escalante J. P., Diez A., Figueroa Casas M. (2015). Postpartum pulmonary hypertension. *Medicina*.

[5] Demerouti E., Manginas A., Rammos S., Athanassopoulos G., Karatasakis G., Pavlides G. (2012). Postpartum pulmonary arterial hypertension: two cases covering a wide spectrum of presentations. *Hellenic journal of cardiology*.

[6] Olsson K. M., Jais X. (2013). Birth control and pregnancy management in pulmonary hypertension. *Seminars in Respiratory and Critical Care Medicine*.

[7] Endorsed by the European Society of Gynecology (ESG), the Association for European Paediatric Cardiology (AEPC), and the German Society for Gender Medicine (DGesGM), Regitz-Zagrosek V., Lundqvist C. B. (2011). ESC guidelines on the management of cardiovascular diseases during pregnancy: the Task Force on the Management of Cardiovascular Diseases during pregnancy of the European Society of Cardiology (ESC). *European Heart Journal*.

[8] Sheppard S. J., Khalil R. A. (2010). Risk factors and mediators of the vascular dysfunction associated with hypertension in pregnancy. *Cardiovascular & Hematological Disorders-Drug Targets*.

[9] Olley P., Whitaker W. (1967). Postpartum pulmonary hypertension. *Obstetrics and Gynecology*.

[10] Weiss B. M., Zemp L., Seifert B., Hess O. M. (1998). Outcome of pulmonary vascular disease in pregnancy: a systematic overview from 1978 through 1996. *Journal of the American College of Cardiology*.

[11] Reinartz P., Nowak B., Wildberger J., Schaefer W., Buell U. (2006). PIOPED II in the diagnosis of pulmonary embolism: evolution or stagnation?. *Journal of Nuclear Medicine*.

[12] Batman P. A., Thomlinson J., Moore V. C., Sykes R. (1998). Death due to air embolism during sexual intercourse in the puerperium. *Postgraduate Medical Journal*.

[13] Lifschultz B. D., Donoghue E. R. (1983). Air embolism during intercourse in pregnancy. *Journal of Forensic Sciences*.

[14] Truhlar A., Cerny V., Dostal P. (2007). Out-of-hospital cardiac arrest from air embolism during sexual intercourse: case report and review of the literature. *Resuscitation*.

[15] Soon E., Treacy C. M., Toshner M. R. (2011). Unexplained iron deficiency in idiopathic and heritable pulmonary arterial hypertension. *Thorax*.

[16] Galiè N., Hoeper M. M., Humbert M. (2009). Guidelines for the diagnosis and treatment of pulmonary hypertension: the task force for the diagnosis and treatment of pulmonary hypertension of the European Society of Cardiology (ESC) and the European Respiratory Society (ERS), endorsed by the International Society of Heart and Lung Transplantation (ISHLT). *European Heart Journal*.

[17] Sandoval J., Gaspar J., Pulido T. (1998). Graded balloon dilation atrial septostomy in severe primary pulmonary hypertension. A therapeutic alternative for patients nonresponsive to vasodilator treatment. *Journal of the American College of Cardiology*.

[18] Keogh A. M., Mayer E., Benza R. L. (2009). Interventional and surgical modalities of treatment in pulmonary hypertension. *Journal of the American College of Cardiology*.

[19] Trulock E. P., Edwards L. B., Taylor D. O. (2006). Registry of the International Society for Heart and Lung Transplantation: Twenty-third Official Adult Lung and Heart-Lung Transplantation Report–2006. *The Journal of Heart and Lung Transplantation*.

[20] Hemnes A. R., Kiely D. G., Cockrill B. A. (2016). Statement on pregnancy in pulmonary hypertension from the Pulmonary Vascular Research Institute. *Pulmonary Circulation*.

[21] Mantha S., Karp R., Raghavan V., Terrin N., Bauer K. A., Zwicker J. I. (2012). Assessing the risk of venous thromboembolic events in women taking progestin-only contraception: a meta-analysis. *BMJ*.

